# A prospective case-control study on miRNA circulating levels in subjects born small for gestational age (SGA) evaluated from childhood into young adulthood

**DOI:** 10.1371/journal.pone.0228075

**Published:** 2020-01-24

**Authors:** Elena Inzaghi, Anna Kistner, Daniela Germani, Annalisa Deodati, Mireille Vanpee, Lena Legnevall, Katarina Berinder, Stefano Cianfarani

**Affiliations:** 1 Dipartimento Pediatrico Universitario Ospedaliero, “Bambino Gesù” Children’s Hospital – Tor Vergata University, Rome, Italy; 2 Department of Molecular Medicine and Surgery, Karolinska Institutet, Stockholm, Sweden; 3 Department of Medical Radiation Physics and Nuclear Medicine, Imaging and Physiology, Karolinska University Hospital, Stockholm, Sweden; 4 Dipartimento di Medicina dei Sistemi, University of Rome Tor vergata, Rome, Italy; 5 Department of Women’s and Children’s Health, Karolinska Institutet and University Hospital, Stockholm, Sweden; 6 Patient Area Endocrinology and Nephrology, Karolinska University Hospital, Stockholm, Sweden; Albert Einstein College of Medicine, UNITED STATES

## Abstract

**Objective:**

microRNAs (miRNAs) associated with metabolic risk have never been extensively investigated in SGA subjects. The aim of the current study was to evaluate miRNAs in SGA and AGA subjects and their relationships with the metabolic status and growth.

**Design and methods:**

A prospective longitudinal case-control study was performed in 23 SGA with postnatal catch-up growth and 27 AGA subjects evaluated at the age of 9 and 21 years. Circulating levels of miR-122-5p, miR-16-5p, miR-126-3p, and miR-486-5p were assessed by qPCR.

**Results:**

SGA subjects were shorter both at 9 and at 21 years. No significant differences in insulin like growth factors and metabolic profile were found with the exception of basal glycemia at 9 years. miRNA levels did not differ between SGA and AGA subjects, at 9 and 21 years. miR-16-5p and miR-126-3p levels were higher at 9 than at 21 years. In SGA subjects, miR-122-5p at 9 years was inversely related to adiponectin levels at 21 years and miR-486-5p at 9 years was inversely related to whole-body insulin sensitivity at 9 years and directly related to Hb1Ac at 21 years. Regression analyses showed no predictive value of miRNAs for growth parameters in neither SGA nor AGA subjects.

**Conclusions:**

SGA with postnatal catch-up growth did not show any difference in metabolic risk markers or miRNA circulating levels compared to AGA controls in childhood and young adulthood. miR-122-5p during childhood could identify SGA subjects at higher risk of developing insulin resistance and, eventually, type 2 diabetes in adulthood but further studies are needed to confirm it.

## Introduction

Early life events are critical for the susceptibility to adult chronic diseases and for adult metabolic outcomes [1] and a suboptimal in utero environment leads to low birth weight and intrauterine growth restriction (IUGR), which have been widely associated with the development of cardiovascular disease, insulin resistance, type 2 diabetes (T2D), hypertension, and hyperlipidemia in adulthood [2, 3, 4, 5, 6, 7, 8]. Small for gestational age (SGA) subjects are a heterogenous population in which the pattern and timing of catch-up growth seem to play a major role in influencing the long-term metabolic risk [9, 10].

The molecular mechanisms underlying the development of IUGR-related metabolic anomalies are unknown, and the involvement of epigenetic mechanisms and the regulation of microRNA (miRNA) expression has been hypothesized [11,12].

MiRNAs are short (~22 nucleotides), non-coding single-stranded RNAs, which post-transcriptionally regulate gene expression by inhibiting translation or promoting degradation of the target messenger mRNA. MiRNAs influence many biological processes, regulate cellular function and are involved in metabolic regulation [13,14]. MiRNA circulating profile differs between physiological and pathological states. As serum levels of miRNAs are stable in clinical samples and resistant to the degradation by ribonucleases, they have been proposed as biomarkers and predictors of diseases, including cardiometabolic disease [15,16, 17, 18]. Furthermore, miRNA expression in placenta has been even associated with prenatal and postnatal growth [19]. Pregnancy-related miRNAs may be involved in pregnancy complications [20]. The intrauterine environment is an established key factor potentially affecting postnatal health and miRNAs may mediate the impact of an adverse uterine milieu on metabolic homeostasis.

We have recently described a specific profile of circulating miRNAs in obese SGA children characterized by upregulation of miR-92a-3p, miR-122-5p, miR-423-5p, miR-484, miR-486-3p and miR-532-5p, and down-regulation of miR-181b-5p [21].

The aim of this study was to assess the circulating levels of miRNAs, previously associated with metabolic alterations [22,23,24,25,26] in SGA and AGA subjects longitudinally evaluated from childhood into adulthood.

## Materials and methods

### Study population

This prospective longitudinal study was performed in a cohort of 23 SGA (13 females/10 males) and 27 appropriate for gestational age (AGA) (16 females/11 males) subjects, longitudinally evaluated at the age of 9 and 21 years.

The initial study population included 26 SGA children born at term and 30 AGA children born at term (control group), evaluated and described in a previous study [27]. These patients were evaluated at the Karolinska Institutet and Karolinska University hospital in Stockholm at mean age 9.8 years (26) and 24 SGA and 27 AGA agreed to participate to a follow up study after puberty. The final population of the present study consists of 23 SGA children and 27 AGA subjects, as one sample of a SGA subject was not available.

Being born SGA was defined as a birth weight less than—2 SD (i.e. below the sex-specific 2.5th percentile for gestational age) according to Swedish reference data. Ultrasound performed early during pregnancy was used to define gestational age. Standard deviation scores (SDS) for height and Body Mass Index (BMI) are based on the Swedish reference curves at 9 years and in adulthood [28].

All participating subjects were singletons born in Stockholm at Karolinska University Hospital, without chromosomal anomalies, congenital infections or life-threatening congenital anomalies. The SGA group consisted of children born from 1990 to 1993 as well as the control group which consists of healthy normal birth weight subjects born from 1990 to 1993. At follow-up, the entire study group was investigated between the 26th of April 2013 to the 1st of March 2014.

The study was approved by the Regional Ethical Review Board, Stockholm. In the prepubertal study the families were contacted and offered to participate in the study by a letter. After receiving the confirmatory response, signed by the parents and the child, the families were called by the research nurse to explain the study more in detail, to confirm participation by oral consent and to arrange a time for the study (prepubertal). In adulthood all participants gave their written informed consent.

At 9 years of age patients were admitted to the hospital after a 10- to 12-h overnight fast. Height and weight were measured and for each patient body mass index (BMI) was calculated by using the following formula: BMI = weight (kg)/height (m2) and expressed as SD.

Fasting blood samples were obtained to evaluate blood glucose, insulin, IGF-binding protein-1 (IGFBP-1), and leptin. A standard oral glucose tolerance test (OGTT) was performed in all children by giving 1.75 g ⁄ kg body weight glucose (up to 75 g). Thereafter, blood samples were taken at 30 and 120 min for measurements of blood glucose, insulin and IGFBP-1. Glucose tolerance was categorized as normal based on 2-h plasma glucose concentration during an OGTT according to reference criteria [29]. Values of homoeostasis model assessment—insulin resistance (HOMA-IR) and of a modified whole-body insulin sensitivity index (WBISI) were used as insulin resistance ⁄ sensitivity markers [30, 31]. The HOMA-IR was calculated as fasting insulin (mU⁄L) · fasting glucose (mmol ⁄L) ⁄ 22.5. WBISI was calculated as 10 000 ⁄ square root of [(fasting glucose · fasting insulin) · (mean glucose · mean insulin during OGTT, using the three values at 0, 30 and 120 min)] [31]. As glucose and insulin measurements were not taken at 60, 90,150 and 180 min, these values are missing in the WBISI calculations.

At 21 years, patients were re-evaluated. Fasting blood samples were obtained to analyse hemoglobin A1C, (HbA1C), blood glucose, insulin, IGFBP-1, lipid profile (cholesterol, triglycerides, HDL-cholesterol (HDL-c), LDL-cholesterol (LDL-c), Apolipoprotein-A1, ApolipoproteinB1), Leptin, and Adiponectin. In addition, waist circumference (WC)/height ratio, WC/hip ratio, fat %, trunk fat % were evaluated. Height was assessed by the same stable length meter. WC and hip ratio were estimated by measuring tape. Fat % and trunk fat % were evaluated by impedance (Body composition analyser BC-418MA, Tanita, Tanita Corporation of America, Inc, Arlington Heights, Illinois, USA).

Serum samples have been collected in 23 SGA and 27 AGA subjects at 9 and 21 years and have been properly stored at– 80°C and used to evaluate insulin-like growth factors (IGF-I and IGF-II) and miRNAs.

### Biochemistry

HbA1c was measured by cation exchange chromatography (MonoS column) with HPLC (Bio-Rad) (Roche Diagnostics, Basel, Switzerland) (normal reference value <5.2%), 9 years. In-house RIA was used for IGFBP-1 with individual serum samples in the same assay. The RIA for IGFBP-1 was performed according to Povoa et al 1984 with intra- and inter-assays CV 3% and 10%, respectively. Blood glucose readings were performed and documented by the nursing staff using HemaCue 201`s method (Hemocue AB, Angelholm, Sweden). In children, commercial kits were used for S-insulin by ELISA (Dako, Glostrup, Denmark). The detection limit was 3 pmol/l. The intra- and interassay CV was 6.7 and 7.5% respectively. In follow-up commercial kits were used for S-insulin by electrochemiluminescence immunoassays (Roche Diagnostics GmbH, Mannheim, Germany). Detectionlimit 0.2–1000 ml/L. Reference value 2–25 ml/L.

Cholesterol and triglycerides were analysed by enzymatic method (dxc800 (2020); Beckman AB, Sychron LX, Beckman Synchron LX, Beckman Coulter Inc., Diamond Diagnostics, Holliston, MA, USA), HDL-c and LDL-c by homogenous method (dxc800 (2020); Beckman AB, Sychron LX, Beckman Synchron LX, Beckman Coulter Inc., Diamond Diagnostics, Holliston, MA, USA). Apolipoprotein-A1 and ApolipoproteinB1 levels were determined by turbidimetry assays (Beckman AB, Sychron LX, Beckman Synchron LX, Beckman Coulter Inc., Diamond Diagnostics, Holliston, MA, USA). For Apo A1, the analysis range was 0.21–3.2 g/L, the reference range for men was 1.10–1.80 g/L, and the reference range for women was 1.10–2.10 g/L. The Apo B analysis range was 0.26–3.2 g/L, and the reference range (<40 years) was 0.50–1.50 g/L.

Leptin was measured by RIA, using HL-81K (Linco Research, Inc., St. Charles, MO, USA). The intra-assay coefficient of variation (CV) for the leptin analysis was 5%. The interassay CV was 4.5%. Adiponectin was analyzed by RIA using HADP-61HK (Linco Research, Inc., St. Charles, MO, USA). The samples are diluted 1:500 before the procedure begins. Analyzing interval Ca 0,8–200 μg/L which corresponds to 0.4–100 mg/L after diluting factorisation, detection limit 1.0 ug/L, reference limit 6,0–25 mg/L. The sensitivity of the adiponectin analysis was 1 mg/l. The intra- and interassay CV were 4 and 8.5% respectively.

### Assays

IGF-I and IGF-II levels have been evaluated on serum samples of all patients at both 9 and 21 years. IGFs have been measured as previously described [32], by using specific immunoassay kit. IGF-I and IGF-II values were expressed as SDS relative to a reference cohort.

### MicroRNA measurement

Circulating RNA was isolated from a serum volume of 100 μl using a miRNeasy Mini Kit according to the manufacturer’s instruction. (Qiagen, Dusseldorf, Germany). Synthetic cel-mir-39 (Applied biosystems, Thermo Fisher scientific, Waltham, MA USA) was used as a control spike-in to help monitor RNA recovery and reverse transcription efficiency. Total RNA was eluted in 14 μl of nuclease free H2O. A fixed volume of 2 μl of RNA eluate was used as input in each reverse transcription reaction using Taqman ADV MicroRNA cDNA synthesis (Applied biosystems, Thermo fisher scientific, Waltham, MA USA) according to the company’s recommendations instructions. Quantitative real-time polymerase chain reaction (qPCR) for miR-486-5p, miR-122-5p, miR-126-5p and miR-16-3p and for miR-484, miR-191, miR-93 and miR-24 were performed by using the Taqman technology (Applied biosystems). A threshold value of 0.2 was set to ensure comparability across plates and a control sample was evaluated in each plates to verify reproducibility. Ct values greater than 32 were excluded. Results were normalized with the mean normalized method [33], which uses the mean of miR-93, miR-191, miR-24 and miR-484 as normalization factor. MiRNA expression levels were calculated according to the ΔCT method.

### Statistics

Results are reported as the mean ± SD. Normal distribution was assessed by Shapiro-Wilk test. Differences between means were assessed using unpaired two-tailed *t* test and one-way ANOVA. The relationships among parameters were evaluated by Pearson correlation. Multiple regression and stepwise regression analyses were used in the selection of predictors of adult height. Significance was assigned for *P* < 0.05. All analyses were performed using SPSS version 21.0 for windows (SPSS, Chicago, Illinois).

## Results

### Anthropometry

SGA subjects, despite having experienced a catch-up growth leading to normal stature, overall remained shorter than AGA subjects, both at 9 (0.08 ± 1.06 vs 0.76 ± 1.2 SDS, p<0.05) and at 21 years (-0.21 ± 0.76 vs 0.65± 1.32 SDS, p<0.05). BMI was not significantly different between SGA and AGA subjects, both at 9 and at 21 years. At 21 years, SGA subjects showed no significant differences in WC/height ratio, WC/hip, total body fat percentage, and trunk fat percentage.

### Biochemistry

Biochemical and metabolic profile were overall not significantly different between SGA and AGA, both at 9 and at 21 years (Tables 1 and 2). Baseline glucose at 9 years, though in the normal range in all children, was significantly higher in SGA subjects (4.62 ± 0.48 vs 4.18 ± 0.53 mmol/L, p<0.01). At 21 years glycemia was not significantly different (4.77 ± 0.29 vs 4.62 ± 0.44 mmol/L, p = 0.16). Insulin was similar between the groups both at 9 (6.17 ± 2.86 vs 8.32 ± 11.19mU/L, p = 0.27) and 21 years (9.07 ± 3.72 vs 9.07 ± 6.64 p = 0.3), as well as HOMA-IR at 9 years (1.27 ± 0.61 vs 1.53 ± 2.03 p = 0.12) and 21 years (1.95 ± 0.86 vs 1.81 ± 1.37 p = 0.23). No significant differences were found in leptin, adiponectin, lipid profile, apolipoprotein A1 and apolipoprotein B1 both at 9 and 21 years.

**Table 1:** Biochemical and metabolic profile in small for gestational age (SGA) and appropriate for gestational age (AGA) subjects at 9 years.

	SGA	AGA
**AGE (YEARS)**	9.8 ± 0.22	9.8 ± 0.31
**HEIGHT SDS**	0.08 ± 1.06	0.76 ± 1.2
**BMI SDS**	- 0.16 ± 0.9	0.27 ± 1.08
**IGF-I SDS**	-1.01 ± 0.6	-1.2 ± 0.58
**IGF-II SDS**	-0.83 ± 0.42	-0.84 ± 0.61
**GLYCEMIA 0’ (MMOL/L)**	4.62 ± 0.48	4.18 ± 0.53
**GLYCEMIA 30’ (MMOL/L)**	7.58 ± 1.23	7.56 ± 1.29
**GLYCEMIA 120’ (MMOL/L)**	5.78 ± 1.04	6.02 ± 0.98
**INSULIN 0’ (MU/L)**	6.17 ± 2.86	8.32 ± 11.19
**INSULIN 30’ (MU/L)**	62.74 ± 30.67	58.85 ± 58.54
**INSULIN 120’ (MU/L)**	32.89 ± 14.91	45.67 ± 34.99
**HOMA-IR**	1.27 ± 0.61	1.53 ± 2.03
**WBISI**	2014.8 ± 476.7	2285.8 ± 874.7
**IGFBP-1 (μG/L)**	44.6 ± 25.3	48.2 ± 27.2
**LEPTIN (μG/L)**	8.37 ± 6.84	8.9 ± 1.14

SDS: standard deviation score. BMI: body mass index. HOMA-IR: homoeostasis model assessment—insulin resistance. WBISI: whole-body insulin sensitivity index

**Table 2:** Biochemical and metabolic profile between small for gestational age (SGA) and appropriate for gestational age (AGA) subjects at 21 years.

	SGA	AGA
**Age (yrs)**	20.09 ± 0.9	20.8 ± 1.1
**Height SDS**	-0.21 ± 0.76	0.65 ± 1.32
**BMI SDS**	- 0.16 ± 0.9	0.27 ± 1.08
**IGF-I SDS**	-1.07 ± 0.6	-1.1 ± 0.5
**IGF-II SDS**	-0.43 ± 0.7	-0.63 ± 0.7
**Glycemia (mmol/L)**	4.77 ± 0.29	4.62 ± 0.44
**Insulin (mU/L)**	9.07 ± 3.72	9.07 ± 6.64
**HOMA-IR**	1.95 ± 0.86	1.81 ± 1.37
**WC/height**	0.46 ± 0.06	0.46 ± 0.05
**Trunk Fat (%)**	19.15 ± 8.75	21.34 ± 10.26
**Leptin (μg/L)**	15.22 ± 13.54	12.22 ± 11.29
**Adiponectin (mg/L)**	8.95 ± 4.05	9.73 ± 7.56
**HbA1c (mmol/mol)**	31.9 ± 2.19	31.96 ± 2.76
**C-Pep (ng/ml)**	0.6 ± 0.2	0.77 ± 0.2
**Cholesterol (mg/dl)**	4.3 ± 0.65	4.4 ± 0.79
**Triglycerides (mg/dl)**	0.85 ± 0.44	0.82 ± 0.46

SDS: standard deviation score. BMI: body mass index. HOMA-IR: homoeostasis model assessment—insulin resistance. WC: waist circumference. HbA1c: hemoglobin A1C. C-pep: c-peptide.

No significant differences were found in IGF-I, IGF-II and IGFBP-1 levels both at 9 and 21 years.

### MiRNAs

**Fig 1:**
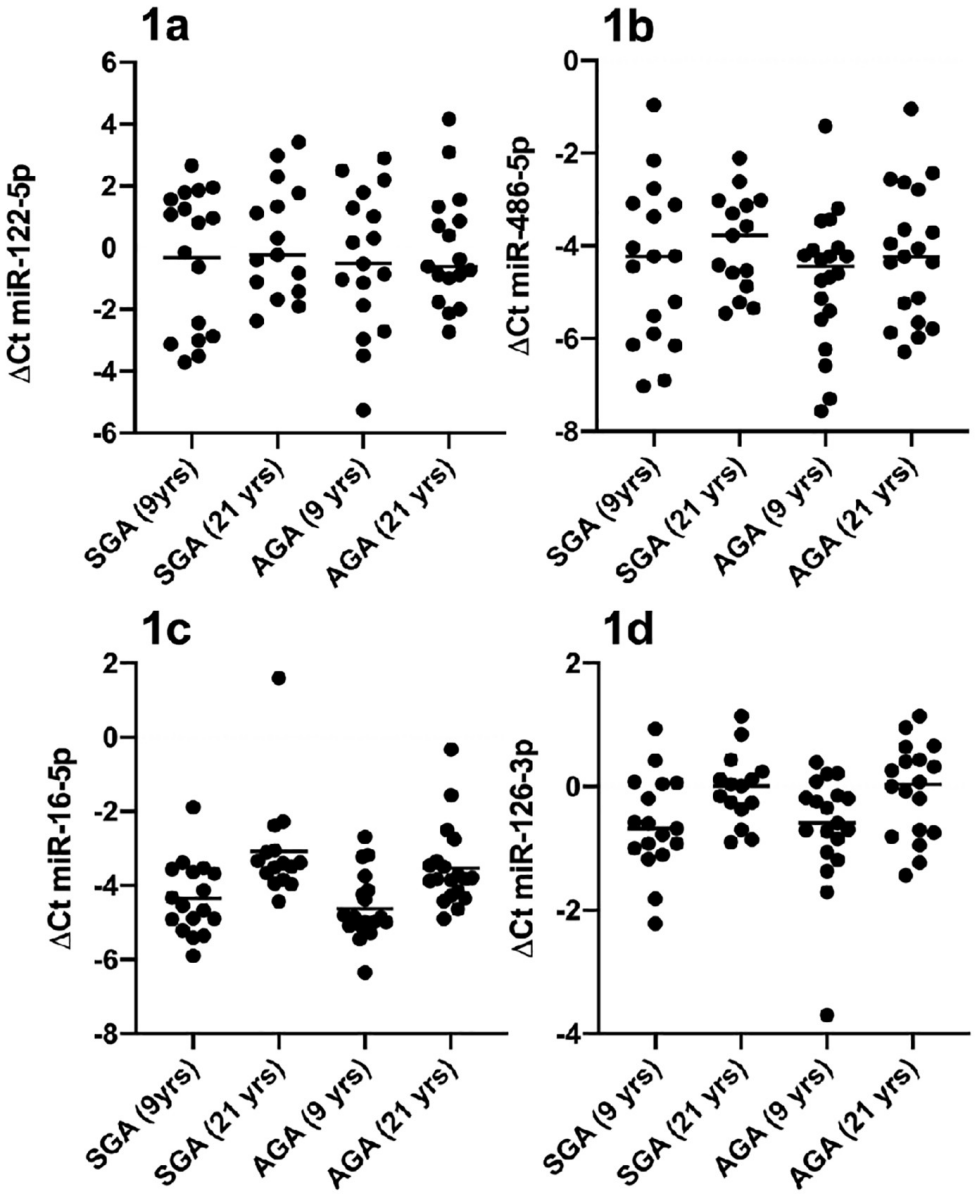
(a-d). MiRNA expression according to age. MiR-122-5p and miR-486-5p levels were not influenced by the age (p > 0.05), while miR-16-5p and miR-126-3p levels were higher at 9 years than at 21 years both in SGA and in AGA subjects (p<0.05). SGA: small for gestational age. AGA: appropriate for gestational age.

MiR-122-5p, miR-16-5p, miR-486-5p and miR-126-3p serum levels were evaluated in all SGA and AGA subjects. MiRNA expression was not influenced by gender. MiR-122-5p and miR-486-5p expression was not influenced by the age (Fig 1a and 1b), whereas mir-16-5p and mir-126-3p levels were higher at 9 than 21 years (Fig 1c and 1d), both in the whole study population and in SGA and AGA groups.

**Fig 2:**
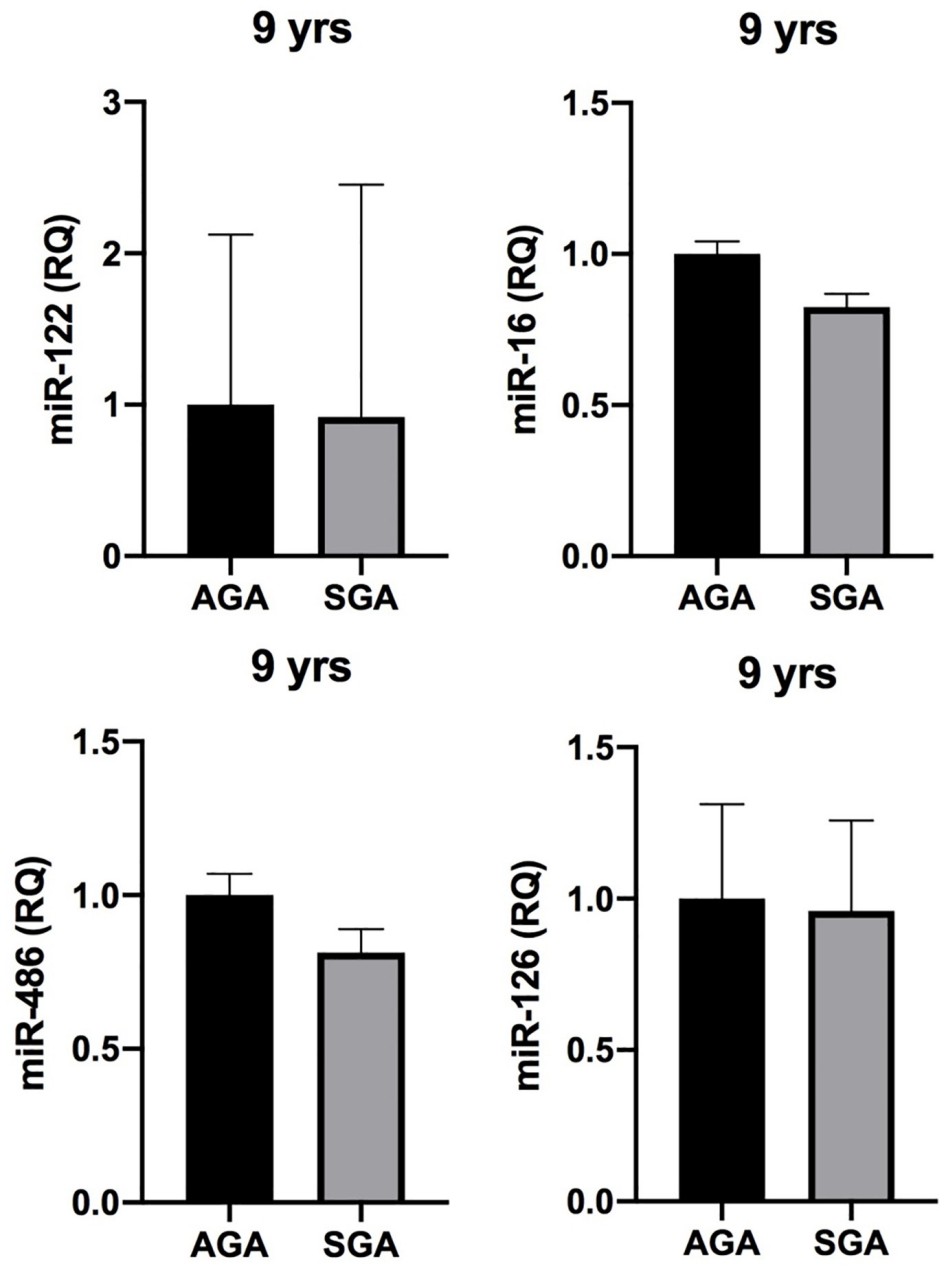
MiRNA expression in SGA and AGA subjects. Comparative analysis of miRNA expression between SGA and AGA subjects at 9 years. SGA: small for gestational age. AGA: appropriate for gestational age. MiR-122: miR-122-5p. MiR-486: miR-486-5p. MiR-16: miR-16-5p. MiR-126: miR-126-3p.

**Fig 3:**
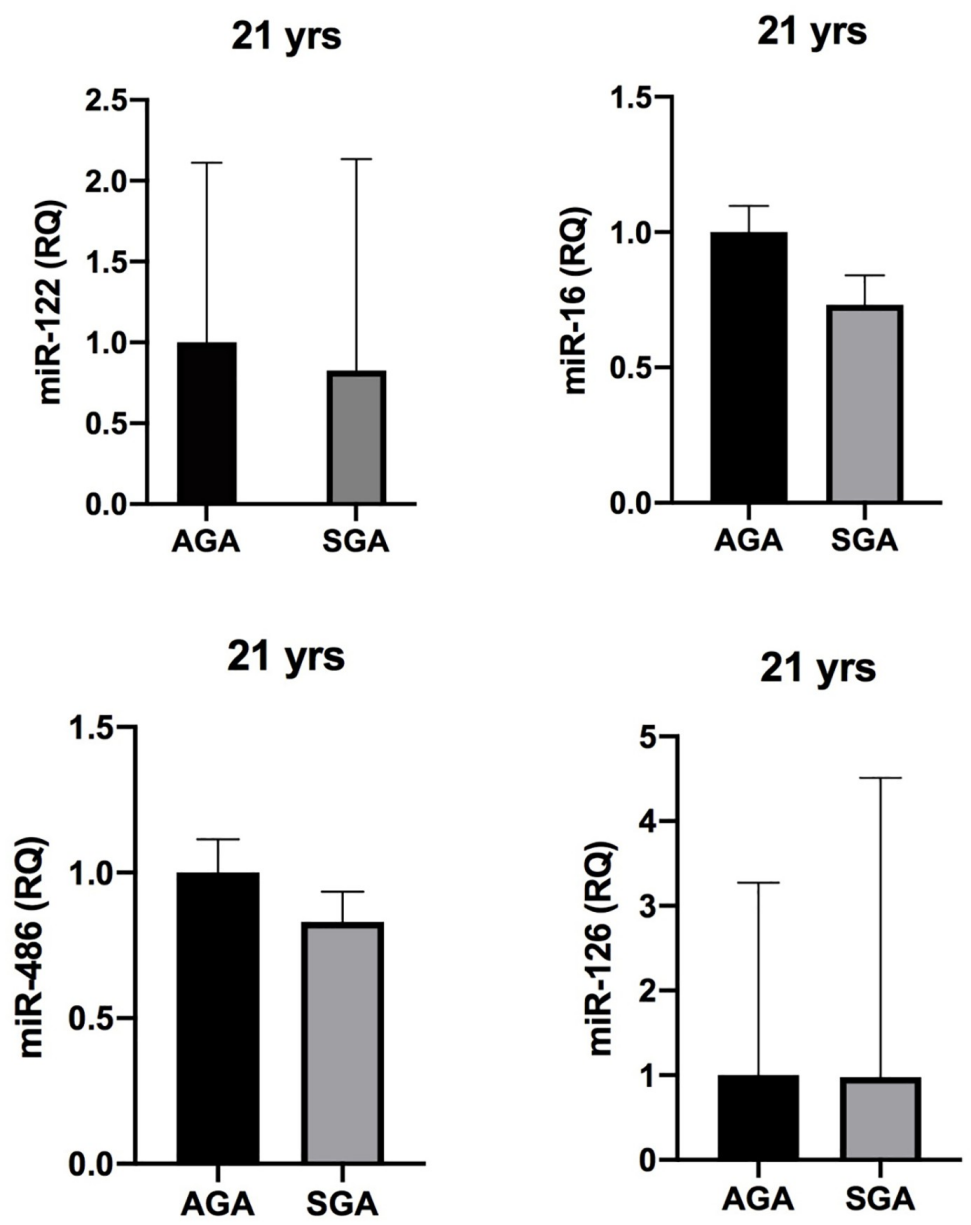
MiRNA expression in SGA and AGA subjects. Comparative analysis of miRNA expression between SGA and AGA subjects at 21 years. SGA: small for gestational age. AGA: appropriate for gestational age. MiR-122: miR-122-5p. MiR-486: miR-486-5p. MiR-16: miR-16-5p. MiR-126: miR-126-3p.

By comparing miRNAs expression between SGA and AGA groups, no significant difference was found in miRNA circulating levels both at 9 (Fig 2) and 21 years (Fig 3).

**Fig 4:**
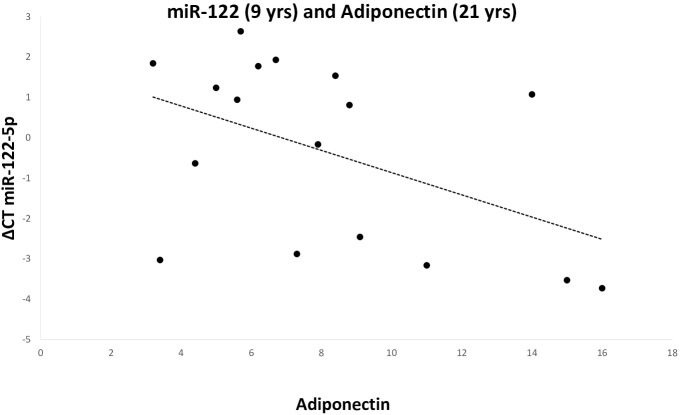
MiR-122 expression and Adiponectin levels. In SGA subjects, miR-122-5p expression at 9 years resulted inversely related to Adiponectin levels at 21 years. SGA: small for gestational age.

**Fig 5:**
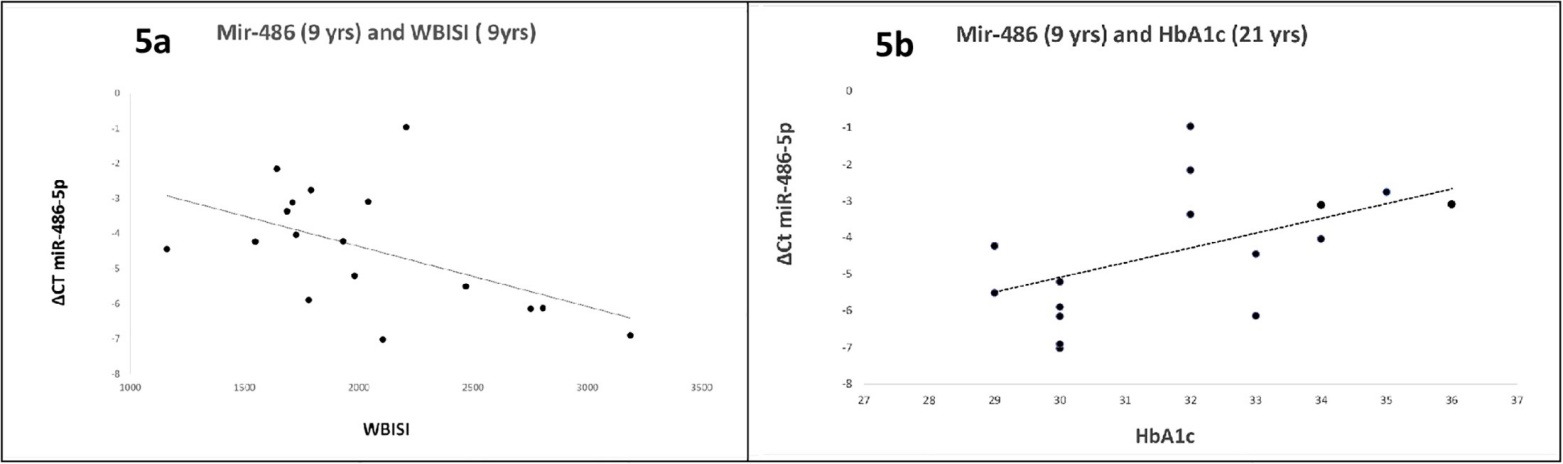
(a and b). MiR-486 expression and WBISI and HbA1c. Mir-486-5p expression at 9 years was inversely related to WBISI at 9 years (5a) and directly related to Hb1Ac at 21 years (5b).

In SGA subjects, mir-122-5p expression at 9 years was inversely related to adiponectin levels at 21 years (r = -0.48, p<0.05) (Fig 4). MiR-122-5p expression was not statistically significantly related with Adiponectin levels at 21 years (r = -0.479, p = 0.071). Mir-486-5p expression at 9 years was inversely related to WBISI at 9 years (r = -0.52, p<0.05) and directly related to Hb1Ac at 21 years (r = 0.52, p<0.05) (Fig 5a and 5b). These associations were not found in AGA subjects.”

### Multiple regression analysis

Stepwise regression analysis showed that no miRNA was predictive of insulin sensitivity (HOMA-IR) at 21 years in both SGA and AGA subjects. The only significant predictor of adiponectin levels at the age of 21 years was mir-122-5p at the age of 9 (β = 0.59, adjusted r^2^ = 0.30, F = 7.43, p<0.02) in SGA subjects, and weight gain between birth and 9 years (β = 0.53, adjusted r^2^ = 0.24, F = 6.0, p<0.03) in AGA subjects. miRNAs were not significant predictors of growth in SGA or AGA subjects. In AGA subjects the best predictors of height at the age of 21 years were father’s height (β = 0.47), birth length (β = 0.47) and mother’s height (β = 0.34), with an adjusted r^2^ = 0.83, F = 26.8, p<0.0001. In SGA children the only significant predictor of height at 21 years was mother’s height (β = 0.60, adjusted r^2^ = 0.32, F = 8.16, p = 0.01).

## Discussion

A suboptimal intrauterine environment leads to low birth weight which has been associated with an increased risk of cardiometabolic disease in adult life [2, 3, 4, 5, 6, 7, 34]. Children born SGA comprise a heterogeneous population with different metabolic and growth outcomes depending on the underlying pathophysiology. Unfortunately, to date, no tools are available to identify subjects deserving a more careful metabolic and growth monitoring and a tailored management.

Although circulating miRNAs have been associated with various metabolic alterations, to our knowledge, this is the first prospective longitudinal case-control study from childhood into early adulthood assessing miRNA profile in SGA subjects. Our results show that SGA subjects who have experienced catch-up growth and are metabolically healthy have circulating levels of specific candidate miRNAs such as miR-122-5p, miR-16-5p, miR-126-3p, and miR-486-5p not different from AGA subjects both in childhood and in early adulthood.

MiR-122 has been widely investigated for its role in metabolism [22]. In adults, circulating miR-122 levels have been associated with the severity of NAFLD, lipid metabolism, obesity, insulin resistance, T2D, and metabolic syndrome [35, 36, 37]. In obese children, serum levels of miR-122 represent an extrahepatic fingerprint of NAFLD [38]. In this study we have shown that the expression of miR-122-5p in childhood is the major predictor of adiponectin levels in early adulthood in SGA subjects. MiR-122 has been correlated with insulin resistance and T2D [39]. As adiponectin levels decrease in case of insulin resistance and are reduced prior to the development of T2D [40], it could be speculated that miR-122-5p levels during childhood could identify SGA subjects at higher risk of developing insulin resistance and, eventually, T2D.

MiR-486-5p is involved in glucose metabolism and insulin signaling [41]. MiR-486-5p increases in obese children [24] and adult patients with T2D and miR-486 has been implicated in accelerating preadipocyte proliferation and myotube glucose intolerance [23]. MiR-486-5p has also been associated with insulin resistance and response to thiazolidenidones [41]. Finally, miR-486 modulates PI3K/Akt signaling by targeting the phosphatase and tensin homolog (PTEN) and the forkhead box O1a (FOXO1A) [42]. Therefore, by modulating these targets, miR-486 may affect insulin sensitivity and blood glucose. Our data support an association between miR-486-5p and glucose homeostasis in SGA subjects as we found an inverse relationship between miR-486-5p expression and index of insulin sensitivity and Hb1Ac levels.

MiR-16 affects insulin signaling and is down-regulated in the skeletal muscle of twins with T2D compared with their non-diabetic identical twins [25]. Placental miR-16 expression predicts the risk of being born SGA [26] and miR-16-5p is down-regulated in IUGR pregnancies [43]. MiR-126 expression is down-regulated in patients with coronary artery disease [44] and miR-126-3p is down-regulated in pregnancies complicated by IUGR [43]. Our results show no significant relationship of miR-16-5p and mir-126-3p with metabolic and growth parameters in both SGA and AGA subjects.

Our data indicate an age dependency of both miR-16-5p and miR-126-3p. This finding is in agreement with the recent observation that some miRNAs are differentially expressed according to age [45] being usually downregulated with increasing age.

During the first years of life most SGA subjects experience a spontaneous catch-up growth and achieve normal height although approximately 10% remain permanently short [46]. All SGA subjects investigated in this study had experienced catch-up growth and achieved a normal height in both childhood and adulthood, though being shorter than AGA subjects.

The metabolic profile of SGA and AGA subjects was essentially similar. The only different parameter, was basal glycemia at 9 years. This is in contrast with previous findings showing that SGA subjects have a worse metabolic profile, especially after experiencing an early catch-up growth [47]. Increased fat mass and abdominal fat have been reported in SGA subjects compared to AGA subjects [48]. Notably, early weight gain rather than birth size influences body composition [49]. Our results show no significant differences in body composition between SGA and AGA subjects. This finding contrasting previous reports may be explained either by the small study cohort or by a different timing of catch-up growth, as we have no information regarding early postnatal growth rate.

IGF system has been associated with metabolism and metabolic parameters. Specifically, IGF-I levels have been related with cardiometabolic risk markers both in children and adults [50], and IGF2 polymorphisms have been related to body weight, insulin sensitivity, fat-free mass, lipid profile, and blood pressure [51]. The results of this study show no differences in IGF system as well as no correlation of IGFs with both anthropometric and metabolic parameters.

We are aware of some limitations of our study. First, in SGA subjects the metabolic risk is deeply influenced by the pattern of early catch-up growth, the faster the early catch-up growth the worse is the impact on cardiometabolic risk [8]. Unfortunately, no information on early growth was available in our subjects. Second, the relatively small sample size may have affected the results. Third, we focused the analysis on candidate mi-RNAs and a complete miRNoma analysis was not performed. Fourth, all SGA subjects investigated in this study had experienced catch-up growth, for this reason our results cannot be applied to SGA subjects without postnatal catch-up growth. Finally, as metabolic alterations may require time to occur, a longer longitudinal study would be more appropriate to test whether miRNAs are predictive of the development of metabolic diseases in adulthood and can be used as early markers of an increased cardiometabolic risk in SGA subjects. The major strengths of this study are the longitudinal prospective design and the original approach applying for the first time miRNA serum profile analysis to SGA subjects.

In conclusion, our data do not support the speculation that SGA subjects have a different/peculiar profile of circulating mi-RNAs related to cardiometabolic risk. Further prospective and more prolonged studies in larger populations of SGA subjects including subjects with both catch-up and no catch-up growth are needed to assess whether miRNAs may represent early biomarkers of long-term increased cardiometabolic risk.

## Supporting information

S1 Dataset Includes Ct values of miRNAs in SGA and AGA subjects. (XLS)
